# Identification of Pepper Leaf Diseases Based on TPSAO-AMWNet

**DOI:** 10.3390/plants13111581

**Published:** 2024-06-06

**Authors:** Li Wan, Wenke Zhu, Yixi Dai, Guoxiong Zhou, Guiyun Chen, Yichu Jiang, Ming’e Zhu, Mingfang He

**Affiliations:** 1 College of Electronic Information & Physics, Central South University of Forestry and Technology, Changsha 410004, China; 20212927@csuft.edu.cn (L.W.); t20060599@csuft.edu.cn (G.Z.); 2 College of Bangor, Central South University of Forestry and Technology, Changsha 410004, China; 20216175@csuft.edu.cn (W.Z.); 20216067@csuft.edu.cn (Y.D.); 3 College of Computer & Mathematics, Central South University of Forestry and Technology, Changsha 410004, China; t20010609@csuft.edu.cn; 4 Hunan Polytechnic of Environment and Biology, Hengyang 421005, China; jiangyichu@hnebp.edu.cn

**Keywords:** pepper disease identification, TPSAO-AMWNet, ARPC, MTDFA, WfrLoss, TPSAO

## Abstract

Pepper is a high-economic-value agricultural crop that faces diverse disease challenges such as blight and anthracnose. These diseases not only reduce the yield of pepper but, in severe cases, can also cause significant economic losses and threaten food security. The timely and accurate identification of pepper diseases is crucial. Image recognition technology plays a key role in this aspect by automating and efficiently identifying pepper diseases, helping agricultural workers to adopt and implement effective control strategies, alleviating the impact of diseases, and being of great importance for improving agricultural production efficiency and promoting sustainable agricultural development. In response to issues such as edge-blurring and the extraction of minute features in pepper disease image recognition, as well as the difficulty in determining the optimal learning rate during the training process of traditional pepper disease identification networks, a new pepper disease recognition model based on the TPSAO-AMWNet is proposed. First, an Adaptive Residual Pyramid Convolution (ARPC) structure combined with a Squeeze-and-Excitation (SE) module is proposed to solve the problem of edge-blurring by utilizing adaptivity and channel attention; secondly, to address the issue of micro-feature extraction, Minor Triplet Disease Focus Attention (MTDFA) is proposed to enhance the capture of local details of pepper leaf disease features while maintaining attention to global features, reducing interference from irrelevant regions; then, a mixed loss function combining Weighted Focal Loss and L2 regularization (WfrLoss) is introduced to refine the learning strategy during dataset processing, enhancing the model’s performance and generalization capabilities while preventing overfitting. Subsequently, to tackle the challenge of determining the optimal learning rate, the tent particle snow ablation optimizer (TPSAO) is developed to accurately identify the most effective learning rate. The TPSAO-AMWNet model, trained on our custom datasets, is evaluated against other existing methods. The model attains an average accuracy of 93.52% and an F1 score of 93.15%, demonstrating robust effectiveness and practicality in classifying pepper diseases. These results also offer valuable insights for disease detection in various other crops.

## 1. Introduction

Since the start of the 21st century, the global cultivation area and yield of pepper have continued to grow, with China becoming one of the world’s largest pepper-producing countries. As an indispensable crop, pepper is not only widely used in daily life but also closely related to human health [1,2]. With the increasing global demand for pepper production and quality, pepper diseases (such as anthracnose, blight, bacterial spot disease, and viral disease) have become one of the main factors restricting their yield and quality [3]. In the past, a number of pepper diseases have caused severe economic losses, such as the pepper mild mottle virus (PMMoV), which can cause yield losses of up to 40% of peppers, and may lead to yield losses of up to 90% when the disease incidence is as high as 100% in places such as the state of Himachal Pradesh, India; pepper blight, which is caused by pathogens that are capable of destroying an entire pepper crop, especially occurring in East Asia, causing severe economic losses [4]; and chili brown spot, a serious disease caused by a fungus that is capable of causing severe losses of an entire chili crop, especially in East Asia, leading to high economic losses [5]. Pepper leaf diseases often directly reflect early growth problems in crops; therefore, the accurate and rapid identification of these diseases is crucial for detecting growth problems in time and taking accurate control measures. Traditional disease identification methods mainly rely on manual observation, which has the problems of a low accuracy of identification and low efficiency and is also susceptible to subjective factors [6]. To cope with the limitations of traditional disease identification methods, researchers have explored various techniques to improve the accuracy and efficiency of pepper disease diagnosis. Early studies mostly relied on feature engineering and machine learning algorithms, such as the use of digital image processing techniques based on color, shape, and texture features combined with traditional classifiers such as support vector machines (SVMs) for disease classification [7]. In recent years, research has shifted to the use of image processing and artificial intelligence techniques for the rapid and accurate diagnosis of pepper diseases. This is essential for the timely detection and effective treatment of plant health problems.

With the significant improvement of computing technology and data processing capabilities, traditional machine learning methods such as KNN [8], Random Forest (RF) [9], and Artificial Neural Networks (ANNs) [10] have been widely used in the field of crop disease recognition. Despite some success, these methods still have limitations, such as the inability to process large amounts of data and a lack of generalization capabilities. In recent years, with the rapid development of computer vision and artificial intelligence, deep learning has emerged as a crucial tool for the rapid diagnosis of agricultural diseases [11,12]. Traditional artificial feature extraction methods have problems of insufficient representations and incompleteness. Through the ability of automatic learning features, deep learning networks have achieved remarkable results in crop disease image classification, greatly improving the accuracy and efficiency of disease recognition, especially convolutional neural networks (CNNs) to automate disease detection [13]. Through deep learning networks, we can train models to automatically extract and learn features. Compared to traditional manual feature extraction methods, deep learning is capable of capturing a wider range of information in images and acquiring more distinguishable feature representations. This greatly improves the accuracy and efficiency of disease diagnosis. In addition, AI technology enables automated disease detection and diagnosis through high-precision image recognition, which has been reported to have an accuracy rate of more than 95%, which is significantly better than that of traditional manual visual assessment methods. AI technology can also predict the risk of disease outbreaks and real-time monitoring, among other things, which demonstrates the potential of AI to enhance the process of disease prevention and precision management decision-making [14]. The amount of crop disease image data is huge, and cannot be processed effectively by traditional methods. With parallel computing capabilities, deep learning models can quickly process a large number of image samples, thus accelerating the process of disease recognition. By learning from large-scale data, deep learning networks can better understand the differences between different diseases and accurately classify them. Meanwhile, AI technology is becoming a key tool for enhancing plant ecological health management. For example, Dai M et al. proposed an enhanced lightweight model based on the GoogLeNet architecture, the cross-validation results showed that the accuracy of the model is 97.87%, but this model only greatly improves its calculation accuracy and performance. Although their model has fewer parameters and a higher efficiency, it is not targeted. There are some shortcomings in its recognition under relatively complex backgrounds [1]; Bhagat, M et al. proposed a method for the disease detection of pepper that combined Local Binary Pattern (LBP) characteristics with visual geometry group network (VGG-16) characteristics and applied only to bacterial spot disease. However, there are still some deficiencies in the recognition of pepper bacterial spot disease with the characteristics of minor diseases [15]; KNuanmeesri, S et al. established a pepper disease diagnosis model by applying filter and wrapper feature selection methods and Multi-layer Perceptron Neural networks (MLPNNs). After selecting key features, the diagnostic model was established by using the selected datasets and applied to MLPNNs. Through cross-validation, the accuracy, precision, and recall of the model reached 98.93%, 98.92%, and 98.89%, respectively. However, the key features of the disease may not be selected by filtering and packaging feature selection methods, so there may be a certain risk of overfitting the model [16]; the methods of Chen, W et al. were conducive to processing pepper images in an HSV color space, enabling a convolutional neural network (CNN) to extract additional features. Compared with the RGB color space, this method improved its accuracy and other indicators, and it was more suitable for diseases with prominent color features, such as viral diseases, but for diseases that do not pay attention to color space, it has certain limitations [17]; Sharma, R et al. proposed the detection of Pepper Leaf Blight Disease (PLBD) in pepper leaves based on the faster region-based convolutional neural network, R-CNN, and a multi-classification method to evaluate the model’s performance, the detection accuracy and multi-classification accuracy were 99.39% and 98.38%, respectively, and the computational efficiency of the model was evaluated. The average inference time of each image was 0.23 s, which was suitable for deployment in high-throughput disease detection applications [18]; Shreyas, K et al. used the Laplacian channel and unsharp covering method to process the image and used the Canny edge method to segment the image, aiming to realize the identification of early diseases and improve the agricultural output. This precise consideration of its local characteristics is beneficial, because it may be the key feature. Thus, the combination of local and global features makes its classification more accurate [19].

**Figure 1:**
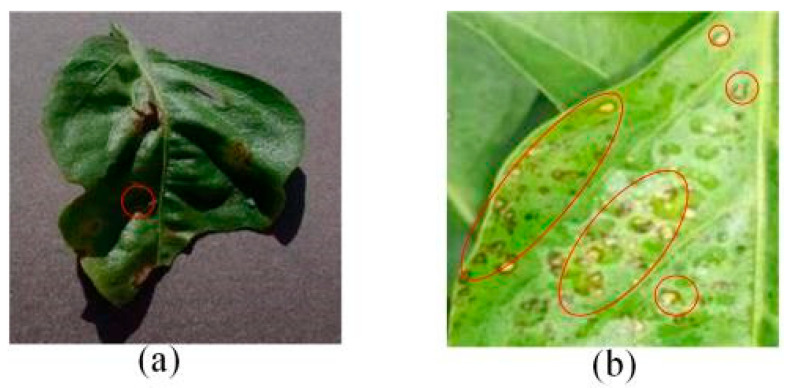
Challenges encountered. (**a**) Refers to the edge-blurring challenge; (**b**) Refers to the challenge of tiny feature recognition.

The above studies provide valuable insights into the agricultural identification of pepper diseases. However, there are still three major challenges in pepper disease identification and classification:

(a) Blurred edge: Pepper leaf images often have the problem of blurred edge details, which may be caused by a variety of factors such as lighting and image resolution. Training directly with these images significantly reduces the ability of the network to extract its disease features, resulting in a decrease in the accuracy of the detection, as illustrated in Figure 1a.
(b) Characteristics of minor diseases: Minor spots or patches appear on pepper leaves, which are often relatively minor and easy to ignore, resulting in the loss of minor but critical feature information, resulting in the model ignoring those regions of disease, so it is challenging to accurately classify them, as illustrated in Figure 1b.
(c) Get the best learning rate: Optimizing the learning rate of convolutional neural networks is a challenging task, as the process is not only time and effort-consuming but also often difficult to ensure the accuracy of the results. This can cause the model to fail to reach its best performance.

To solve the problem of blurred image edges, the accuracy of image recognition is improved; Chen, X et al. [20] used binary wavelet transform combined with Retinex (BWTR) to de-noise and enhance images, remove noise points and edge points, and retain important texture information. Then, the Both-channel Residual Attention Network model (B-ARNet) was used to identify the image, and the overall detection accuracy was about 89%. Zhang, Y et al. [21] used the Asymptotic Non-local Means algorithm (ANLM) to reduce image noise interference and reduce the difficulty of tomato leaf disease feature extraction in the recognition network. Then, a Multi-channel Automatic Orientation Recurrent Attention Network (M-AORANet) was proposed to extract rich disease features, extract multi-scale fine features, and recycle them, and its recognition accuracy reached 96.47%. Begum, S et al. [22] improved the contrast-limited adaptive histogram equalization (ICLAHE) technique to enhance image quality, and then used kernelized gravity-based density clustering (KGDC) to segment the lesion area. Finally, gated self-attention convolutional MobileNetV3 (GSAtt-CMNetV3) was used for feature extraction and classification, and the recognition accuracy reached 97.87%. Yang, M et al. [23] extended the task set through a data augmentation module to enhance the robustness to noise and perspective changes and then mapped the samples to an embedding space with strong intra-class similarity and inter-class divergence, constructed a multi-layer graph attention network (GAT), and updated according to a new hybrid loss to obtain the classification results, and a higher accuracy than that of the typical FSL method was obtained. Yang, W et al. [24] proposed a DEGREE network, which enhanced the edge retention capability of the super-resolution reconstruction process, in the recurrent recovery process, and LR images and their edge mapping could collectively be used to infer the sharp edge details of HR images.

To solve the problem of the loss of key feature information with minor disease features, Zuo, X et al. [25] explored the modeling of semantic relations by pixel-level spatial self-attention and then used block-level channel self-attention to enhance the feature discrimination ability of different crop varieties. Finally, a spatial reasoning module was used to conduct the sequential modeling of spatial geometric relations of image patches. The classification accuracies of the three datasets were 88.32%, 89.95%, and 89.75%, respectively. Luo, W et al. [26] learned fine-grained features by enhancing the sub-feature semantics of global features, arranged the feature channels of a CNN into different groups to achieve sub-feature semantics and enhance the distinguishability of sub-features, and guided the groups to be activated at highly distinguishable target locations by using weighted combination regularization. Du, R et al. [27] adopted a progressive training strategy to effectively integrate features of different granularities and select consistent block convolution to encourage the network to learn features of the same category at a specific granularity. Liu, H et al. [28] proposed the hierarchical stage feature aggregation (HSFA) module and the feature abstraction (FFA) module, which were used to cascade multi-layer features to aggregate the multi-scale information of bird images and extract invariant clues of birds based on feature selection based on the differentiation score, respectively. The recognition accuracy rates on the two datasets were 91.0% and 90.9%, respectively. Yang, G et al. [29] proposed an attention mechanism that effectively utilized image information regions, and used transfer learning to rapidly construct multiple fine-grained image classification models for crop diseases, achieving good classification results with F1 values as high as 93.05%.

Addressing the challenge of identifying the optimal learning rate, Loizou, N et al. [30] introduced a stochastic Polyak step-size (SPS) for setting the learning rate in stochastic gradient descent (SGD), aiming to achieve a swiftly converging adaptive learning rate. Meanwhile, Melinte, D et al. [31] implemented a periodic learning rate strategy, enabling the learning rate to fluctuate within predefined upper and lower bounds. This technique allows for improved classification accuracy in training without the need for further adjustments. Golovko, V et al. [32] incorporated the ReLU activation function utilized within the neural network model to calculate an adaptive learning rate. This approach was specifically designed to autonomously determine the optimal step size for reducing the objective function of the neural network.

Recent research shows that deep-learning techniques have made significant progress in pepper disease identification. Nonetheless, several challenges remain, especially in dealing with fuzzy edges, subtle disease features, and enhancing model generalization to avoid overfitting. In view of this, we propose a new method: a TPSAO-AMWNet model based on enhanced ResNeXt-50 to identify pepper diseases. The contributions of this paper are outlined as follows:

1. An AMWNet method is proposed to effectively address the issues of edge ambiguity and minor disease characteristics:
  (a) This paper proposes an Adaptive Residual Pyramid Convolution (ARPC) structure, which solves the problem of fixed scale limitation and insufficient feature transfer in traditional pyramid convolution by combining residual connection and adaptability; the Squeeze-and-Excitation (SE) module is also used to highlight pepper disease feature image areas in order to more accurately highlight the edges of pepper disease.
  (b) This paper proposes Minor Triplet Disease Focus Attention (MTDFA), by combining two-space and two-channel processing, it effectively balances the extraction of global and local features, and solves the limitation of traditional attention mechanisms in capturing features of minor diseases; this method strengthens the local detail capture of pepper leaf disease characteristics, while maintaining the attention to global characteristics, enhancing the feature expression, and reducing the interference of unrelated regions, thus improving the accuracy and robustness of pepper disease classification.
  (c) In this paper, we propose a hybrid loss function combining Weighted Focal Loss and L2 regularization (WfrLoss), which can be optimized for multiple targets at the same time, aiming to balance the model’s fitting ability and generalization potential. By adjusting $\alpha_{t}$ weights, WfrLoss effectively deals with the class imbalance and difficult-to-classify samples and reduces the risk of overfitting by introducing L2 regularization. Adjusted β values balance the weights between Weighted Focal Loss and L2 regularization terms to adjust model training strategies. This method not only improves the performance of the model on training data but also enhances the generalization ability of the model.
2. A tent particle snow ablation optimizer (TPSAO) algorithm is proposed. The algorithm is initialized with tent chaotic mapping to generate more diverse and uniformly distributed initial solutions. The algorithm combines the advantages of particle swarm optimization (PSO) and the snow ablation algorithm (SAO). By integrating the strengths of these two algorithms, the particles are able to strike a balance between local and global optimal solutions, thereby significantly enhancing the search capability and convergence speed of the algorithm. Upon the application of this algorithm, it becomes possible to more accurately determine the optimal learning rate, effectively addressing issues in the learning rate optimization process, and greatly improving overall model training efficiency.
3. In this study, our proposed the TPSAO-AMWNet model accurately determines the optimal learning rate using the TPSAO optimization algorithm and achieves an average accuracy of 93.52% and an F1 score of 93.15% on homemade datasets. This method is effective in recognizing pepper leaf disease images with blurred edges and efficiently extracting and identifying micro-disease features. In addition, this technique not only classifies pepper disease images quickly and accurately but also provides valuable insight into the application of deep learning in precision agriculture and field disease classification.

## 2. Materials and Methods

### 2.1. Data Acquisition and Expansion

In order to optimize, debug, and evaluate the performance of pepper disease image recognition and classification algorithms, it is necessary to provide appropriate datasets. To solve this problem, we collect data, mainly from the public data of the Pepper Disease and insect pest Image Recognition Challenge on the Kaggle platform (https://www.kaggle.com (accessed on 14 January 2024)) and public data PlantVillage sets (https://aistudio.baidu.com/datasetdetail/224113 (accessed on 15 January 2024)), and we screen their images, obtaining a total of 1876 images from PlantVillage and 2334 images from Kaggle to create pepper disease datasets. The images in the public datasets have different dimensions, including 1279 × 1706 pixels, 887 × 1920 pixels, 2736 × 3648 pixels, and 256 × 256 pixels.

To build adequate training datasets and avoid image duplication, we adopt a variety of data augmentation techniques, including removing duplicate images, applying horizontal and vertical flips, and random cropping, to improve the diversity of the gathered image data. The datasets contain 6315 images with a training set to test set ratio of 8:2. Table 1 summarizes the number and proportion of pepper disease photos by category. The datasets cover 5 types of pepper disease symptoms, including Anthracnose, Phytophthora blight, Healthy, Bacterial spot, and Mosaic virus, as shown in Table 2, which reflects the appearance and characteristics of pepper disease images. All are saved in a jpg format.

In the training set’s data-processing stage, the given pepper leaf images are randomly cut to different sizes and aspect ratios, then the cropped images are scaled to specified dimensions, the given image is randomly rotated horizontally with a given probability (default is 0.5) to further data enhancement, and finally the image is converted into a tensor and normalized. In the validation set’s data-processing stage, the input pepper leaf image is converted into an input feature map of 256 × 256 and then trimmed in the middle area of the image to ensure the consistency and efficiency of data input. This procedure not only enhances the model’s generalization capabilities but also lays solid groundwork for future performance assessments.

### 2.2. TPSAO-AMWNet-Based Pepper Disease Recognition

In this study, we propose a novel TPSAO-AMWNet convolutional neural network model based on ResNeXt-50, which aims to detect pepper diseases quickly and accurately in natural environments. This model combines ARPC, MTDFA, and WfrLoss, and employs the TPSAO optimization algorithm to fine-tune the learning rate, enhancing the training outcomes. Through these improvements, the TPSAO-AMWNet improves the efficiency of generalization and classification, ensures that the disease can be accurately identified even in the presence of blurred edges or small disease features, and effectively avoids the problem of overfitting.

**Figure 2:**
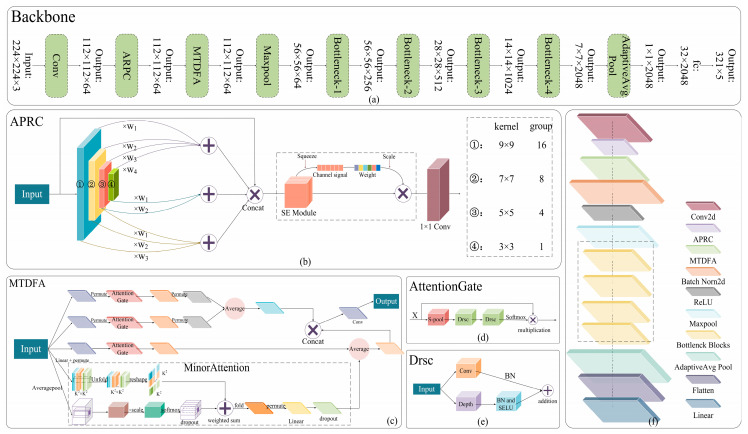
A diagram of the structure of the TPSAO-AMWNet. (**a**) represents the backbone network; (**b**) represents the APRC structure; (**c**) represents the MTDFA structure model; (**d**) represents the components of the AttentionGate module; (**e**) represents the components of the Drsc convolution; (**f**) shows the AMWNet model structure.

The overall structure of the TPSAO-AMWNet model is illustrated in Figure 2. The TPSAO-AMWNet model comprises a convolutional layer, ARPC, MTDFA, and multiple bottleneck block modules. The first convolution layer of the model uses 7 × 7 convolution to extract image features initially, and then uses ARPC for multi-scale and cross-level adaptive feature extraction, and integrates SE, which helps to capture edge feature information and solve edge-blurring problems. Then, MTDFA is used to strengthen the processing of input feature map features. By applying the methods of an attention module, feature mapping, and unfolding in multi-channel and multi-space, the model can deal with the extraction of minor disease features, which can highlight the attention to a pepper disease with minor disease feature areas, so the detection accuracy of the model is thereby improved. Following that, there are four bottleneck blocks, which serve as the fundamental modules in the TPSAO-AMWNet. Each bottleneck block consists of three convolutional layers, a corresponding batch normalization layer, a ReLU activation function, and a residual connection. The three convolutional layers process the input feature maps. The batch normalization layer adjusts the mean of the data to 0 and the variance to 1, thus helping to stabilize the training process. The ReLU activation function introduces nonlinear characteristics so that the network can learn more complex feature representations. Finally, WfrLoss serves as the loss function of the TPSAO-AMWNet and helps prevent overfitting while improving the generalization ability of the model.

#### 2.2.1. Adaptive Residual Pyramid Convolution (ARPC)

Convolution is a fundamental neural network operation that can filter the features of the receptive field and extract the most prominent feature from this region [33]. In the pepper disease image classification task, the convolution operation can extract various local features of the image, such as the edge, texture, and color, which are crucial for disease classification [34]. However, images of pepper diseases are often characterized by multi-scale features, and traditional single-scale convolution operations may not adequately capture information at all scales. The advantage of pyramid convolution lies in the ability to perform convolution operations at different scales to generate feature maps with different resolutions, so as to fully understand the image content. Therefore, opting for pyramid convolution in the classification of pepper disease can effectively capture multi-multiscale feature information and enhance the accuracy and robustness of the classification process. Nonetheless, pyramid convolution also has some shortcomings: the first is the limitation of its fixed scale, which may lead to the insufficient performance of the model in handling the image features of different scales and complexities, especially in capturing edge and detail information, and the second is its insufficient feature transmission, wherein the characteristic information may gradually decay during the transmission process, resulting in the model being unable to effectively use the shallow edge and detail information at a deeper level. Therefore, to overcome these problems, an Adaptive Residual Pyramid Convolution (ARPC) is proposed, and Figure 2b shows its structure. ARPC significantly improves the performance and efficiency of the model in the task of pepper disease classification by combining residual connection and adaptability; the introduction of residual connection not only enhances feature transmission and extraction but also allows the network to transmit feature information across layers, thus deepening the understanding of the image. In addition, its adaptability enables the model to dynamically adjust the feature extraction method according to different scales and complexities, reduces the requirements of manual parameter adjustment, helps to capture the details and edge information in the image, and reduces edge blur. In ARPC, residual connectivity aids in capturing global features and also allows for the integration and fusion of local features across various scales, thereby boosting the ability to understand the overall structure and semantic content of the image.

We divide its input into four branches, each with a convolutional kernel of different sizes for capturing information at different scales. First, feature information at different scales is extracted by four convolutional kernels of different sizes, each of which is of size 3 × 3, 5 × 5, 7 × 7, and 9 × 9, so that local detail information, larger region information, and the largest range of global information features can be captured. Each branch is composed of a pyramid convolutional layer (PyConv) and a residual convolutional layer, where the PyConv layer uses grouped convolution with different numbers of groups, 1, 4, 8, and 16, respectively, for feature extraction at different scales. Then, an adaptive weight parameter is introduced. After *softmax* normalization, the weight of each branch is automatically adjusted according to the distribution of the data, as shown in Equation (1).

(1) $$adaptive\_weights=softmax(W)=[\frac{e^{\omega_{1}}}{\sum_{i=1}^{4}e^{\omega_{i}}},\frac{e^{\omega_{2}}}{\sum_{i=1}^{4}e^{\omega_{i}}},\frac{e^{\omega_{3}}}{\sum_{i=1}^{4}e^{\omega_{i}}},\frac{e^{\omega_{4}}}{\sum_{i=1}^{4}e^{\omega_{i}}}]$$

Next, the output features of each branch are adaptively multiplied and added with the corresponding weights layer by layer to obtain the fused feature representation, and then the output features of the output are obtained via residual connection with its residual convolution layer, as shown in Equation (2).

(2) $$\begin{array}{c} output=[combined_{1},combined_{2},combined_{3},residual\_conv(x)]\\ combined_{1}=\omega_{1}conv_{1}(x)+\omega_{2}conv_{2}(x)+\omega_{3}conv_{3}(x)+\omega_{4}conv_{4}(x)\\ combined_{2}=\omega_{1}conv_{2}(x)+\omega_{2}conv_{3}(x)+\omega_{3}conv_{4}(x)\\ combined_{3}=\omega_{1}conv_{3}(x)+\omega_{2}conv_{4}(x) \end{array}$$

where $\omega_{i}$ refers to the adaptive weight vector, i∈1,2,3,4, which determines the importance of each convolution branch, and *softmax* is able to convert each element of the vector to non-negative values and the sum of the normalized elements to 1.

In addition, we introduce the SE module [35] to enhance the network’s attention to key features, highlight the edge, and detail information of the image to improve the clarity and contrast of the edges and reduce edge blur. The SE module consists of two main parts: Squeeze and Excitation; in the Squeeze part, the feature map of each channel is compressed into a scalar to capture the global information of each channel. In the Excitation section, the feed-forward neural network is utilized to acquire the importance weights of each channel and to subsequently apply these weights to the original feature map to improve the attention given to important features; eventually, these weights are normalized to the range of 0 to 1 by the Sigmoid function to ensure that they can be considered as valid attention weights, as shown in Equation (3). Finally, the channels are compressed and reduced via 1 × 1 convolution to adjust the number of feature channels to 64 to ensure the consistency of the output features.

(3) $$SE(X)=\sigma (W_{2}\cdot ReLU(W_{1}\cdot AdaptiveAvgPool(X)))$$

where X represents the feature graph of the adapted residual pyramid convolution output, $W_{1}$ and $W_{2}$ represent the weight matrix of the two convolutional layers, respectively.

In summary, this model combines feature information at different scales and is represented by adaptive weights and SE to optimize feature fusion to improve model performance. In Section 3.4.1 of this paper, we provide a detailed analysis of the experimental evaluation of ARPC and the experimental data for module comparison.

#### 2.2.2. Minor Triplet Disease Focus Attention (MTDFA)

Some pepper leaves have characteristics of minor diseases, for which the traditional model is less efficient, as this feature is more difficult to extract, so the ability to more accurately identify and extract these subtle features is crucial for pepper disease identification. The attention mechanism, inspired by human visual processes, is central to more accurately extracting specific features in neural networks using a series of attention-weight assignment coefficients [36]. Attention mechanisms have been widely integrated into a variety of neural network architectures, including graph convolutional neural networks (GCNs) [37], generative adversarial networks (GANs) [38], and convolutional neural networks (CNNs) [39].

With the continuous update and iteration of the attention mechanism, the traditional attention mechanism faces certain limitations. These conventional mechanisms usually focus on the entire feature map or larger regions, thus ignoring some important details, especially when performing poorly when dealing with minor disease features. To overcome these limitations, we propose a novel attention module, the MTDFA module. This module has significant advantages in handling minor features, diversified features, and balancing global and local features. It can not only effectively extract minor disease features, but also has high adaptability and robustness; its structure diagram is shown in Figure 2c.

MTDFA is composed of deep residual separable convolution (*Drsc*), AttentionGate, MinorAttention, *S-Pool* layer, and the *softmax* function, among others. Through the synergistic action of these components, the modules can improve the feature expression of disease images, helping the model to obtain more accurate and comprehensive disease feature information, while reducing the interference of unrelated regions. Specifically, *Drsc* enhances feature representation reduces information loss, and reduces computational complexity; the *S-Pool* layer combines max pooling and soft pooling to extract richer feature information and enhance the ability of the model to capture features of different scales. With the AttentionGate module, by applying attention weights, the important features are highlighted and the unimportant information is suppressed to improve the model’s attention to disease features. The MinorAttention module captures the locally important features in the input feature map through the steps of feature mapping, expansion, attention map generation, weighted feature aggregation, reconstruction, and output projection, which strengthens the spatial perception ability and local detail capture of pepper leaf disease features of the model, thereby improving the accuracy and robustness of pepper disease classification. The *softmax* function ensures that the input and output are smooth and continuous, thus improving the classification performance of the model.

MTDFA is considered from the channel and space perspective, in terms of the channel, focusing on the Channel–Width direction (*CW*) and the Height–Channel direction (*HC*) of the input feature map, respectively; by applying the AttentionGate module on the channel dimension, models are able to more effectively discriminate between important features in different channels, and thus, to better capture the disease-associated features. This focus on the channel helps the model when processing images with complex channel correlations, such as the pepper leaves under different disease states, improving its recognition ability. In terms of space, by applying the AttentionGate module on the *HW*, the model can more effectively focus on the disease-related spatial area in the image; applying the MinorAttention on a local window captures the local spatial information in the input feature graph, and this local attention helps the model focus on minor areas of the image associated with the disease; it combines the attention output from the channel and space, taking advantage of attentional mechanisms in different directions. Through this fusion, the model enables a more comprehensive understanding of the input feature graph, considering both inter-channel relationships and focusing on spatial details, thus improving the identification and classification performance of the model for pepper diseases.

The input feature size of MTDFA is 112 × 112 × 64 (input tensor, $x\in {\mathbb{R}}^{B\times H\times W\times C}$). Then, dual channel and dual space are used to process the input feature map to focus on the regions associated with the disease; specifically, dual channel refers to the direction of the input feature through two different channels, *CW* and *HC*. Before applying AttentionGate, the input feature map is first permuted to adapt to the different attention directions.

In the *CW* direction, the input feature graph is first permuted from (*B*, *C*, *H*, *W*) to (*B*, *H*, *W*, *C*), focusing on the features of the channel and width direction, and is then passed into the respective AttentionGate for processing the output as shown in Equation (4).

(4) $$X_{CW}=\mathrm{AttentionGate}(x_{perm1})$$

In the *HC* direction, the input feature graph is permuted from (*B*, *C*, *H*, *W*) to (*B*, *W*, *H*, *C*) to focus on the features of height and channel direction, and the displaced feature graph is then passed to the respective AttentionGate for processing the output as shown in Equation (5).

(5) $$X_{HC}=\mathrm{AttentionGate}(x_{perm2})$$

where $x_{perm1}$ and $x_{perm2}$ represent the permutation transformation of x in different dimensions.

In the AttentionGate module, the input feature graph is first compressed by the S-Pool layer, which combines maximum pooling and soft-weighted pooling to capture key features and retain certain spatial information, as shown in Equation (6); then, the compressed feature map uses *Drsc* to further extract the compression features, as shown in Equation (7), and at the same time, to keep the computational efficiency. Then, again, it uses *Drsc* to process the compressed features and generate the attention weight but does not use the activation function, to maintain the linear characteristics of the attention weight. Finally, the generated attention weights are normalized by the *softmax* function to ensure that the weight and 1, the normalized attention weights with the original input features and the weighted input feature diagram, allow the network to concentrate on the task-relevant critical areas. This process makes the AttentionGate module able to effectively focus on the input features, highlight the important features related to the task, the generate the overall output expression as shown in Equation (8).

(6) $$S\text{-}Pool=Concat(max(x),\sum_{i\in R}\frac{e^{a_{i}}}{\sum_{j\in R}e^{a_{j}}}*a_{i})$$

where *max* represents the maximum pooling, with ∑ representing the soft pooling.

Deep residual separable convolution (*Drsc*):

(7) $$Drsc=Conv_{point}(Conv_{depth}(x))+Shortcut(x)$$

*Shortcut* represents a shortcut connection, used to match the dimensions, which is a convolution and BN normalization composition.

AttentionGate:

(8) AttentionGate=x⊙softmax(Drsc(S-pool(x)))

where ⊙ means the element-by-element multiplication.

Specifically, dual-space processing refers to applying the AttentionGate module in the Height–Width (*HW*) direction of the input feature map to concentrate on the global spatial details, and implementing the MinorAttention module on the local window of the input feature map to gather intricate local features. The expression of the AttentionGate module applied in the *HW* direction is shown in Equation (9).

(9) $$X_{HW}=\mathrm{AttentionGate}(x)$$

In the MinorAttention module, the input feature graph x is the first feature mapping through a linear layer *v_pj*, through the permute operation to generate a new feature representation *v*($v\in {\mathbb{R}}^{B\times W\times H\times C}$), that improves the expression ability of the feature, whose expression is as shown in Equation (10); then, the *Unfold* operation is used to expand the features of *v*, to obtain a feature representation of the local region, with a local window size of 3 × 3, which is equivalent to a local window scan on the feature map to capture the local features.

(10) $$V_{Unfold}=Unfold(v\_pj(x)),V_{unfold}\in {\mathbb{R}}^{B\times num\_heads\times (h\times w)\times (kernel\_size^{2})\times head\_dim}$$

where *Unfold* represents the operation to expand the feature map *v* into local region features, *H* and *W* are the height and width of the feature map after the *Unfold* operation, *num_heads* is the number of attention heads and is 4, *head_dim* is the dimension of each attention head, and *kernel_size*×*kernel_size* is the size of the local window.

Next, the pooling operation of the input feature graph x is performed to reduce the spatial dimension, and the Attention map is generated by mapping the pooled features through the linear layer *attn*. As shown in Equation (11), its size is ${\mathbb{R}}^{B\times num\_heads\times (h\times w)\times (kernel\_size^{2})\times (kernel\_size^{2})}$, where *kernel_size×kernel_size* is the local window size. Then, the Attention map is scaled and *softmax* is operated to obtain normalized attention weights, whose expressions are shown in Equation (12). These weights are weighted to aggregate local information on local features *v*, highlighting important features and suppressing unimportant features. Finally, the Fold operation is utilized to reconstruct the original spatial dimensions. The final output feature expression is obtained through the linear layer *proj* on the reconstructed features, and random discarding using Dropout is employed to enhance generalization ability. This process enables the MinorAttention module to effectively capture and focus on the locally important features of the input feature map, improving the performance of the model in processing visual tasks.

(11) Attention map=attn(Pool(x))

(12) Attention map=softmax(scale×Attention map)

(13) $$Y=proj(Flod(\mathrm{Attention}\;\mathrm{map}⊗V_{Unfold})),Y\in {\mathbb{R}}^{B\times C\times H\times W}$$

where *Fold* represents the operation of recombining the weighted local regional features into a feature graph, ⊗ represents the weighted aggregation, $x\in {\mathbb{R}}^{B\times H\times W\times C}$, the scale is a scaling factor, *softmax* is used to normalize the weights, and *proj* represents the final feature mapping performed through a linear layer.

Finally, the attention output from the channel and space is fused to utilize the advantages of attention mechanisms in different directions. Specifically, the module first uses three AttentionGates (focusing on Channel–Width, Height–Channel, and Height–Width direction, respectively) and one MinorAttention (focusing on features in the local window) to process the input feature map, so as to obtain four groups of attention-weighted feature maps. Then, the feature maps from *CW* and *HC* are fused to obtain *x_fusion1*, whose expression is shown in Equation (14); at the same time, the feature maps from *HW* and MinorAttention are fused to obtain *x_fusion2*, whose expression is shown in Equation (15). Following this, *x_fusion1* and *x_fusion2* cascade and form a comprehensive feature representation called x_fused, considered to be different from spatial direction and local window attention, then x_fused, feature fusion, and dimension reduction by convolution to get the final output features. The feature can be used for pepper disease classification and subsequent tasks, with its expression shown in Equation (16). Through this process of fusion and cascading, the MTDFA can provide a rich and discriminative feature representation, considering both the relationship between channels and focusing on the local detail features in space, to balance global and local features and further improve the identification and classification performance of the model for pepper diseases.

(14) $$X_{fusion1}=0.5\times (X_{cw}+X_{hc})$$

(15) $$X_{fusion2}=0.5\times (X_{hw}+Y)$$

(16) $$y=Conv(Concat(X_{fusion1},X_{fusion2}))$$

where *Concat* represents the characteristic cascade, and *Conv* represents the convolution operation.

In the above process, using AttentionGate and MinorAttention, the model considers both the relationship between channels and the spatial features, which pays special attention to the extraction of minor features of pepper diseases; this makes the feature extraction of minor disease images more accurate and improves the detection and identification accuracy of the model. In Section 3.4.2 of this paper, we provide a detailed analysis of the experimental evaluation of MTDFA and experimental data for its module comparison.

#### 2.2.3. Weighted Focal and L2-Regularized Mixed Loss Function (WfrLoss)

In the field of deep learning, the loss function plays a crucial role in quantifying the disparity between the model’s predicted outcomes and the actual labels. As a result, it guides the adjustment of network parameters and facilitates optimization [40]. Particularly in image classification tasks, the loss function is vital as it determines how well the model conforms to the training data and represents a primary goal in optimizing the model [41]. Therefore, the loss function is an integral component in the training process of neural network models. According to its experimental data characteristics and experimental tasks, Weighted Focal Loss (*WFL*) is selected, which can handle the category imbalance and the difficult-to-classify sample [42] well. We designed a mixed loss function composed of Weighted Focal Loss and L2 regularization, simply called WfrLoss. Relative to conventional loss functions, WfrLoss boosts the model’s learning efficiency with datasets by including L2 regularization. This method effectively moderates the balance between the model’s fitting prowess and its generalization skills by adjusting its weight parameters, thereby reducing overfitting and enhancing its effectiveness on new data. Moreover, precise adjustments to the weight of the L2 regularization term refine the model’s learning approach for superior performance. Adjusting the weight β balance to the combination of the Weighted Focal Loss and the L2 regularization allows for the adjustment and control of the model training tactics based on complexity. Finally, the Weighted Focal Loss and the L2 regularization term loss are combined to obtain the overall loss value, as shown in Equation (19). This approach successfully enhances the model’s generalization capacity by balancing prediction accuracy with model complexity, thereby efficiently preventing the issue of overfitting.

(17) $$WFL(p_{t})=-\alpha_{t}{\left(1-p_{t}\right)}^{\gamma }\log(p_{t})$$

(18) $$L2=\beta *\left\|Inputs\right\|\_2$$

(19) $$Loss=Loss_{WFL(p_{t})}+L2$$

$p_{t}$ represents the probability that the model predicts that the sample belongs to the true class. $\alpha_{t}$ is a weight term used to adjust the importance of difficult and easy samples. γ is an adjustable parameter used to adjust the loss scaling of hard and easy samples. Since it is a multiclass classification task, t denotes the category.

As shown in Equation (19), the WfrLoss consists of the Weighted Focal Loss and L2 regularization functions. The Weighted Focal Loss is a commonly used loss function for classification problems. It is utilized to quantify the discrepancy between predicted values and actual labels, effectively addressing issues such as class imbalance and difficult-to-classify samples. Therefore, we introduce the weight $\alpha_{t}$ of each category according to the data characteristics. In this experiment, we set the following according to the proportion of the category data: the weight of 20% is set to 1, the weight of 18% is set to 1.3, and the weight of 19% is set to 1.2. Such settings can better handle class imbalance and difficult-to-classify samples. The γ is the loss scaling for the difficult adjustment sample, which is set to 2. If the γ is 0, the loss function represents the cross-entropy loss function. L2 regularization helps alleviate the overfitting problem of the model and prevents it from overfitting the training data. By limiting the size of the model parameters, L2 regularization reduces model complexity and thus improves its generalization ability. In WfrLoss, L2 regularization is used to impose constraints on the norm of the model input via the β that regulates the degree of regularization.

WfrLoss, more suitable for classification and identification tasks than for dealing with category imbalance and difficult-to-classify samples, can optimize multiple targets simultaneously. Section 3.4.3 of this paper specifically analyzes WfrLoss to further verify its superiority in the multi-task scenario.

#### 2.2.4. Tent Particle Snow Ablation Optimizer (TPSAO)

In general, determining the appropriate learning rate can improve the convergence speed of the model and improve its precision and ability to generalize. The traditional practice usually involves manually tuning parameters and conducting multiple training sessions to find the best learning rate, which is time-consuming and may lack precision. To address these challenges, optimization algorithms are introduced to automatically identify the most suitable learning rate, thereby avoiding tedious manual trials and adjustments. This automated technique significantly reduces both time and labor while also decreasing the chances of errors associated with the manual adjustment of parameters.

In 2023, the snow ablation optimizer (SAO) was proposed as an innovative intelligent optimization algorithm, inspired by the sublimation and melting process of snow. During melting, snow could be converted into liquid water, and by sublimation, snow was converted directly into steam [43]. In addition, liquid water could also be converted into steam through evaporation; these two physical processes, melting and sublimation, give the SAO a strong optimization ability, fast convergence rate, and high accuracy. Meanwhile, the SAO overcomes problems such as group diversity and convergence non-equilibrium. However, although the SAO performs well in the search for local optimal solutions, there are deficiencies in finding globally optimal locations. The problem of its being easy to fall into the local optimal solution may affect the effectiveness of the model’s training. To address this problem, we propose a combinatorial approach for the particle snow ablation optimizer (PSAO). Meanwhile, we note that the SAO adopts the traditional random initialization method in the initialization stage, which may lead to an uneven distribution of the initial solutions, limiting the extensive exploration of the algorithm in the search space. Therefore, we introduce the tent chaotic mapping as an improvement measure for the initialization phase. The tent chaotic map generates more diverse and uniformly distributed initial solutions that aid the algorithm in a more extensive search. The unpredictability and ergodic properties of the chaotic maps effectively prevent the algorithm from falling into the local optimal solution in the early stage and improve its global search capability [44]. This approach not only enhances the robustness of the algorithm but also improves its ability to tackle complex multi-spike problems. It addresses the issue that traditional random initialization methods may lead to inefficient search and limited exploration capabilities. In conclusion, we propose an innovative intelligent optimization algorithm for the tent particle snow ablation optimizer (TPSAO), the problem of the local optimal solution, and the uneven distribution of the initial solution.

Like the SAO, the TPSAO also uses mathematical modeling to simulate two physical processes that achieve its academic ablation—melting and sublimation. This includes random initialization, exploration, development, and the two-population mechanism. However, unlike the SAO, the TPSAO first improves in the random initialization stage to the tent chaotic map initialization stage, so as to generate a more diverse and evenly distributed initial solution; in addition, PSO technology is adopted in the position update strategy in the development stage to enhance the global search ability of the algorithm, thereby reducing the likelihood of the situation of falling into the local optimum and ensuring the effective retrieval of the global optimum. The position update in the TPSAO algorithm can be explained by Equations (20) and (21).

(20) $$x_{i}(t+1)=\left\{\begin{array}{c} Elite(t)+RB_{i}(t)⊗(r_{1}\times (G_{i}(t)-X_{i}(t))+(1-r_{1})\times (\bar{X}-X_{i}(t))),i\in index_{a}\\ M\times G_{i}(t)+RB_{i}(t)⊗(r_{2}\times (G_{i}(t)-X_{i}(t))+(1-r_{2})\times (\bar{X}-X_{i}(t))),i\in index_{b} \end{array}\right.$$

(21) $$X_{i}(t+1)=x_{i}(t+1)+V_{i}(t+1)$$

Elite(t) includes randomly selected individuals from several elite groups in the group, as shown in Equation (22); G(t) represents the current optimal solution, $X_{\sec ond}(t)$ and $X_{third}(t)$ represent the second best and the third best individuals in the current population, respectively; $X_{c}(t)$ represents the position of the centroid of the fitness values ranked in the top 50% of the individuals, for whose mathematical expressions, as shown in Equation (23), $N_{1}$ indicates the number of leaders, that is, $N_{1}$ is equal to half the size of the whole swarm, and $X_{i}(t)$ represents the ith best leader; M is the melting rate, which is a key parameter to simulate the melting behavior in the development stage, as shown in Equation (24); $t_{\max}$ is the termination condition; T(t) is the average daily temperature; RB(t) represents a vector containing random numbers, based on a Gaussian distribution, representing the Brownian motion, as shown in Equation (25); the sign ⊗ represents entry-wise multiplications; $r_{1}$ represents the randomly chosen numbers from the [0, 1]; $r_{2}$ represents a randomly selected number from [−1, 1]; $\bar{X}$ represents the centroid position of the whole population, with the corresponding mathematical expression, as shown in Equation (26); and due to the dual-population mechanism, we denote the whole population and these two subpopulations as *N*, $N_{a}$, and $N_{b}$, respectively. Among them, $N_{a}$ is responsible for the exploration, and is assigned to perform the exploitation. Then, in subsequent iterations, the size of $N_{b}$ gradually declines and the size of $N_{a}$ is accordingly increased. $index_{a}$ and $index_{b}$ denote a set of indexes including line numbers of individuals in $N_{a}$ and $N_{b}$ in the entire position matrix.

(22) $$Elite(t)\in [G(t),X_{\sec ond}(t),X_{third}(t),X_{c}(t)]$$

(23) $$X_{c}(t)=\frac{1}{N_{1}}\sum_{i=1}^{N_{1}}X_{i}(t)$$

(24) $$M=(0.35+0.25\times \frac{e^{\frac{t}{t_{\max}}}-1}{e-1})\times T(t),T(t)=e^{\frac{-t}{t_{\max}}}$$

(25) $$f_{RB}(x;0,1)=\frac{1}{\sqrt{2\pi }}\times \exp(-\frac{x^{2}}{2})$$

(26) $$\bar{X}(t)=\frac{1}{N}\sum_{i=1}^{N}X_{i}(t)$$

In the TPSAO, the iterative process first starts with randomly generated populations, as shown in Equation (27), which produces a matrix of size *N* × *dim*. Then, based on the initial solution of its population, the tent chaotic map is combined to generate the initial population solution, as shown in Equation (28). Finally, the initial population solution based on the tent chaotic map is obtained, as shown in Equation (29). Then, there is the initialization of the swarm positions, velocities, best fitness, Objective_values, Elite, Global_Best_Position, best position, and Global_Best_Fitness.

(27) $$x=L_{b}+rand(N,dim).*(U_{b}-L_{b})$$

(28) $$f(x,\alpha )=\left\{\begin{array}{cc} \frac{2x}{\alpha } & 0\leq x<\alpha \\ \frac{2(1-x)}{1-\alpha } & \alpha \leq x\leq 1 \end{array}\right.$$

(29) $$\begin{array}{c} x_{i+1,j}=f(x_{i,j},a),i=1,2,\cdots ,N\\ X=x_{i+1,j}*U_{b} \end{array}$$

*N* is the population size, α is the parameter of the tent chaos map, *j* and dim are the representative dimensional size, $L_{b}$ is the lower bound representing the population, and $U_{b}$ is the upper bound representing the population.

The algorithm enters the loop iteration phase, during which the following operations are performed on each particle in turn:

(a): Calculate the individual optimal position for each particle: $c_{1}r_{1}(p_{i}(t)-y_{i}(t))$, where $c_{1}$ is the weight of the individual’s best position.
(b): Calculate the global best position: $c_{2}r_{2}(G_{i}(t)-y_{i}(t))$, where $c_{2}$ is the weight of the global best position.
(c): Update the speed, as shown in Equation (30), where *w* is the inertial weight, $c_{1}$ is the individual learning factor and $c_{2}$ is the influence of the social learning factor, the inertial weight determines the degree of the inertia of the particle in the search space, the higher value is conducive to jumping out of the local optimal solution but may lead to search instability, and the lower value is conducive to the convergence to the global optimal solution. The individual and social learning factors control the degree of particle updates according to the individual and group optimal solution, balancing local and global search. At the same time, skip the optimal solution by using the maximum speed limit to prevent particles from moving too fast, limiting the range as shown in Equation (31).
  (30) $$V_{i}(t+1)=wV_{i}(t)+c_{1}r_{1}(p_{i}(t)-y_{i}(t))+c_{2}r_{2}(G_{i}(t)-y_{i}(t))$$
  (31) $$V_{i}=clip[V_{i},L_{b}-X_{i},U_{b}-X_{i}]$$
(d): Update the location, $X_{i}(t)=x_{i}(t)+V_{i}(t)$, achieved by constantly adjusting the velocity and position of each particle. In each iteration, the velocity vector of the particle is updated according to parameters such as the individual and population optimal solutions and inertial weights, and then the position of the new velocity vector. In this way, the particles constantly move in the search space to find the optimal solution.
(e): At each iteration, the current position of each particle is verified to determine if a new individual optimum has been reached. If the position is better than the previously recorded optimal position, it is updated. In addition, it also checks whether this position is the current global optimum, and updates the global best record after confirmation. This process helps to optimize the learning and decision efficiency of the whole network.

**Figure 3:**
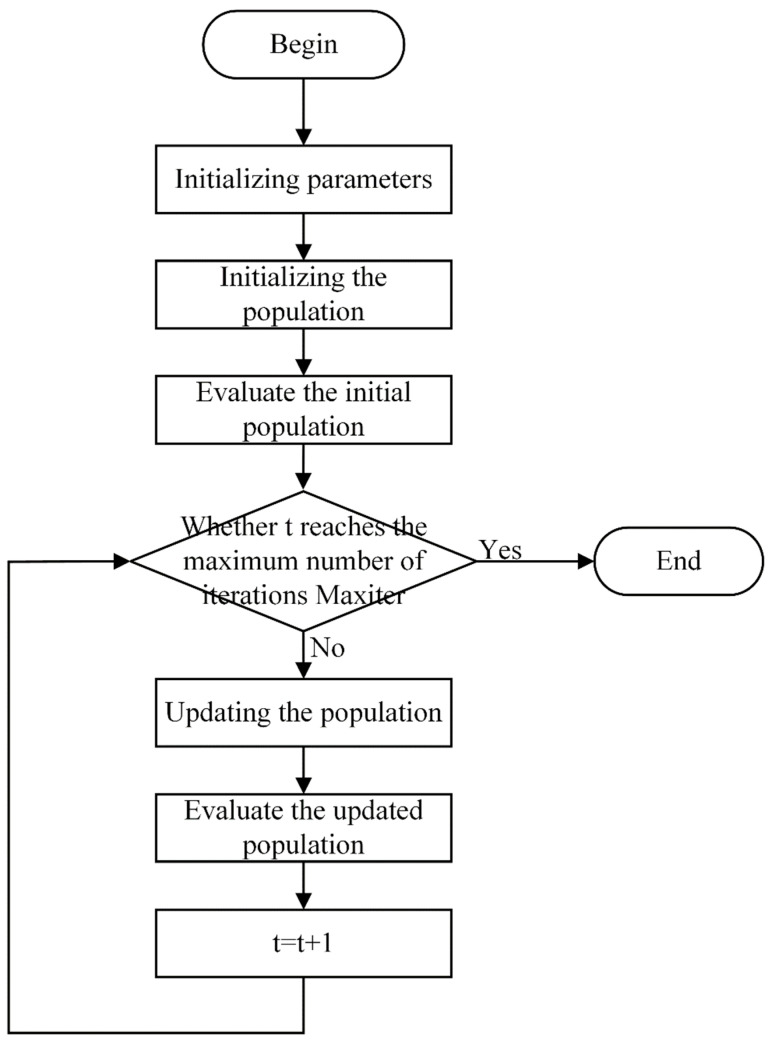
A flowchart of the TPSAO’s execution.

The TPSAO algorithm enhances its global search function by making the particles guided by both the individual and the global best position, thus improving the efficiency of finding the best learning rate. In order to enhance the repeatability of the TPSAO algorithm, we have written the pseudocode of the algorithm in detail and made the corresponding execution flow chart, which is shown in Algorithm 1 and Figure 3, respectively. This combination of a pseudocode and a flowchart not only improves the clarity of the description, but also enables the reader to understand the specific details of the implementation more accurately, thus effectively improving the transparency and operability of the algorithm. In Section 3.4.4 of this paper, we further validate the effectiveness of this algorithm by conducting a detailed analysis of the TPSAO’s performance through module comparison experiments.
**Algorithm 1:** The pseudocode for the TPSAO (Tent Particle Snow Ablation Optimizer).**Input:** t,${\;t}_{max}$ = Maxiter, *N*,${\;N}_{a}=N_{b}=\frac{N}{2}$,${\;c}_{1}$,${\;c}_{2}$, w, dim, upper_bound, lower_bound, α**Output:** Global_Best_Position, Global_Best_Fitness**Begin**   Initialization of the swarm positions, velocities, best fitness, Objective_values, Elite, Global_Best_Position, best position, Global_Best_Fitness   Fitness evaluation   Record the current G(t) and Update Elite, Objective_values   **while**$\;(t\;\text{<}\;t_{max}$) **do**    Calculate the snow melt rate M,
$X_{c}(t)$
    Randomly divide the whole population N into two subpopulations
$N_{a}$ and
$N_{b}$
    **for** each individual **do**       Calculate each individual’s position        Update position and velocities     
**end for**
    Fitness evaluation    Update Global_Best_Position, Global_Best_Fitness    t = t + 1
   **end while**
   Return Global_Best_Position, Global_Best_Fitness**end**

## 3. Experimentation and Analysis

### 3.1. Experimental Environment

The datasets for the experiment are categorized into five groups: Anthracnose, Phytophthora blight, Healthy, Bacterial spot, and Mosaic virus, totaling 4210 images. The pepper disease images are all standardized to a size of 256 × 256 to ensure consistency. After data augmentation, there are 6315 pictures of pepper disease, which are divided into training and test sets at an 8:2 ratio. The experiments are conducted on a Northwest Area B/421 machine provided by the AutoDL platform’s cloud server. Comprehensive details of the experimental configuration are presented in Table 3. The experiments in this work are all conducted on the same hardware and software platform, and Table 3 shows the hardware and software configurations being tested. Because the optimal combination of hyperparameters depends not only on the model but also on the hardware and software environment, the hyperparameters of each network are uniformly designed to avoid the influence of hyperparameters on the experimental results. Table 4 presents the hyperparameter settings.

### 3.2. Experimental Indicators

To accurately evaluate the performance of the model, we use several key metrics: the F1 score, precision, recall, and average accuracy. The relevant evaluation metrics are calculated as follows:

(32) $$Precision_{i}=\frac{TP_{i}}{TP_{i}+FP_{i}}$$

(33) $$Recall_{i}=\frac{TP_{i}}{TP_{i}+FN_{i}}$$

(34) $$F1\text{-}score_{i}=2\times \frac{precision_{i}\cdot Recall_{i}}{precision_{i}+Recall_{i}}$$

(35) $$Prec=\frac{\sum_{i=1}^{n_{c1}}Precision_{i}}{n_{c1}}$$

(36) $$Rec=\frac{\sum_{i=1}^{n_{c1}}Recall_{i}}{n_{c1}}$$

(37) $$F1=\frac{\sum_{i=1}^{n_{c1}}F1-score_{i}}{n_{c1}}$$

(38) $$AAC=\frac{1}{n_{c1}}\sum_{i=1}^{n_{c1}}\frac{n_{ij}}{n_{i}}$$

where *Prec* represents precision, *AAC* stands for the average accuracy and *Rec* indicates recall. The parameter *i* donates the class index, $n_{c1}$ signifies the total number of instances, with *n* being the number of distinct classes. The variable $n_{ij}$ denotes the count of correctly predicted initial classes, where *j* refers to the prediction outcome and *i* represents the class index. The variable $n_{i}$ signifies the number of initial classes that are accurately predicted.

### 3.3. Experimental Performance Analysis

**Figure 4:**
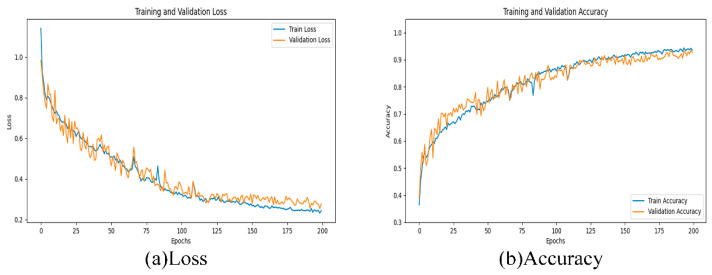
The training loss and accuracy curves.

**Figure 5:**
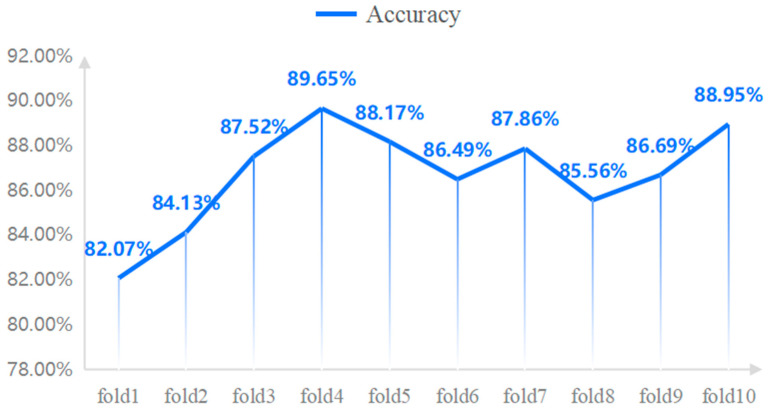
The results of the fold-crossover experiment.

In this paper, the TPSAO-AMWNet model is trained on pepper disease datasets, and Figure 4 shows the loss and accuracy during the training process. The graph clearly shows a steady improvement in the accuracy of the model. After finding the best learning rate via the TPSAO method, we run 200 training runs and achieve the test set average accuracy of 93.52%. To evaluate the performance of the model more comprehensively, we apply the K-fold cross-validation technique to divide the original data into 10 groups. Each set of data is used as the validation set, the rest of the data is used for training, and the process was repeated 10 times. We record and maintain the best result from each cross-validation, and then calculate the average of these results from 10 runs to determine the overall classification accuracy of the network model. Throughout this process, we have kept other hyperparameters fixed, including the batch size of 32 and the learning rate of 0.001, and we have used the Adam optimizer. Finally, we obtain the performance results of the model using a 10-fold cross-validation, as shown in Figure 5.

### 3.4. Effectiveness Analysis of Individual Modules

In this paper, four modules are proposed: ARPC, MTDFA, WfrLoss, and the TPSAO. To verify the effectiveness of these four methods, first, we compare the ARPC module with Pyramid convolution and the TPSAO module. Then, we compare MTDFA with different attention mechanisms such as HaloAttention, CBMA, and Polarized Self-Attention. In addition, we compare the WfrLoss with varying functions of loss, Cross Entropy, and Poly Loss and other optimization algorithms such as SAO and RIME. With the same parameters, we verify the feasibility of this method by adding different modules to the ResNeXt-50 model.

#### 3.4.1. Effectiveness of ARPC

In this paper, ARPC is proposed to finely adjust the feature map, dynamically extract the disease in different scale images, and integrate SE to emphasize the feature image area of pepper diseases, solving the problem of edge blur in pepper diseases. In order to verify its effectiveness, we choose to compare it with this module, without this module, and with pyramid convolution [45]. The experiments are shown in Table 5. The results show that after the ARPC module and the pyramid convolution module are, respectively, added to the ResNeXt-50 network, excellent results are achieved in pepper disease classification. The pyramid convolutional module is appreciated for its straightforwardness, adaptability, and efficient computation. However, it faces challenges such as gradient vanishing or inadequate feature propagation, limiting its effectiveness in addressing edge-blurring issues in pepper disease images. Therefore, it is insufficient compared with ARPC. If the ARPC module is not added, more features are easily lost, resulting in insufficient feature extraction. Therefore, adding the ARPC module is far better than not adding this module. In conclusion, our proposed ARPC module is superior to the pyramid convolutional module. Thus, we choose to add the ARPC module to the input after the first convolution processing, thereby improving the performance and efficiency of the model in the pepper disease classification task.

#### 3.4.2. Effectiveness of MTDFA

In this study, we propose a novel attention module, MTDFA, designed to enhance the feature expression of pepper disease images and improve the performance of the model in extracting minor disease features, thus significantly improving its identification accuracy. To verify the effectiveness of MTDFA, we compare it with three existing attention mechanisms: HaloAttention [46], the CBAM [47], and Polarized Self-Attention [48], and the experimental results are shown in Table 6. Specifically, HaloAttention effectively improves the efficiency of processing large-sized images by focusing on the local “halo” area and processing images through overlapping block structures; however, this method is sensitive to the adjustment of hyperparameters (such as the block size and halo size), and optimizing these parameters needs to be done with higher precision; compared with MTDFA, HaloAttention involves more hyperparameters and the adjustment process is more complicated. By combining spatial and channel attention mechanisms, the CBAM effectively enhances the expression of features and increases the focus of the model on critical information; although CBAM has the advantage of emphasizing local features, it sometimes excessively ignores the importance of the global context. Compared with MTDFA, the CBAM has shortcomings in balancing the processing of local and global information, which may lead to a decrease in its recognition accuracy. Polarized Self-Attention emphasizes a clearer discrimination between key features and background noise by learning extreme (extremely important or unimportant) features; this approach performs well in handling complex scenarios with distinct features; however, its poor performance and may over-highlight certain features while ignoring other equally important details. Polarized Self-Attention has more limitations in adaptability and detail capture compared to MTDFA. According to the experimental results in Table 6, MTDFA outperforms HaloAttention, the CBAM, and Polarized Self-Attention in all indexes. Therefore, we choose MTDFA as the core attention mechanism of the TPSAO-AMWNet to ensure the high efficiency of the model in handling the pepper disease classification task.

#### 3.4.3. Effectiveness of Wfrloss

**Figure 6:**
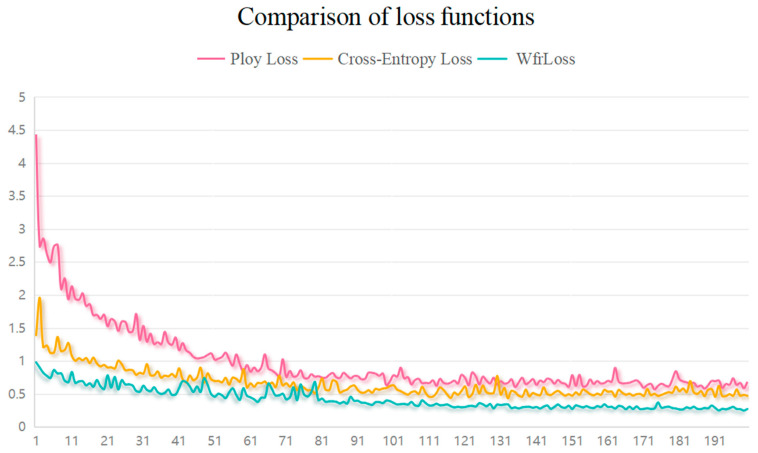
The loss value comparison results. The OX axis represents the number of training rounds, and the OY axis represents the loss value.

In this study, the proposed loss function Wfrloss combines the Weighted Focal Loss and L2 regularization functions, which allows us to balance the focus on classification accuracy and model complexity. By adjusting $\alpha_{t}$, the model cannot affect the classification accuracy of the imbalance of the datasets, and at the same time, by adjusting the β value, it not only helps to balance the classification accuracy and model complexity but also effectively prevents the model from problems caused by overfitting, thereby enhancing the model’s ability to generalize. To verify its validity, we compare it with Poly Loss [49] and Cross Entropy. The experimental results are shown in Table 7, and the changes in the loss values are shown in Figure 6. The experimental results show that Wfrloss is better than Poly Loss and Cross Entropy, and the loss value is lower and more stable, which is very good for the identification of pepper diseases. In bulk, although Poly Loss has an advantage in dealing with class imbalance, its excessive focus on difficult samples may lead to the model overfitting on the training data. Although Cross Entropy Loss helps to directly optimize the probability distribution to improve the classification accuracy of multi-class problems, it is very sensitive to the problem of class imbalance and prone to overfitting when the model is too complex, which may reduce the generalization ability of the model. By contrast, Wfrloss has a clear advantage in preventing overfitting due to its introduced regularization term, as shown by the loss value curve in Figure 6. In view of the above analysis and experimental results, we decided to adopt Wfrloss as the loss function of the TPSAO-AMWNet model. This choice aims to obtain a better training effect and stronger generalization performance to improve the application effect of the model to the pepper disease datasets.

#### 3.4.4. Experiments on the Effectiveness of the TPSAO

The study highlights the main task of determining the optimal learning rate for the training effect and performance of the TPSAO-AMWNet model. Selecting an appropriate learning rate is crucial for achieving the best training results. If the learning rate is too low, it may slow down the training process and fail to reach the global optimum. Conversely, a too-high learning rate may disrupt training stability and negatively impact model effectiveness. Therefore, the accurate calibration of the learning rate is essential to enhance model convergence, ensure stability, and quickly attain optimal solutions, particularly for complex challenges such as pepper disease detection. The TPSAO algorithm is proposed with a tent chaotic map used for initialization, and particle swarm optimization combined with a snow ablation algorithm to rapidly determine the optimal learning rate in the model training process. The initial solutions generated by this method are more diverse and evenly distributed, improving the balance between the individual optimum and the global optimum which significantly enhances the searchability and convergence speed of the algorithm. Unlike traditional adaptive algorithms, the TPSAO not only expands the search scope but also effectively avoids approximate optimal solution traps to identify true optimal learning rates for better model training results. In order to verify the effectiveness of the TPSAO, we conduct a comparison experiment by choosing a common learning rate of 0.001 and comparing it with the learning rate obtained from the original SAO. In this experiment, the parameters of the SAO, the RIME [50], and the TPSAO are set with the number of particles *N* = 50, the maximum number of iterations Maxiter = 100, the upper limit of the learning rate upper_bound = 0.01, and the lower limit of the learning rate lower_bound = 0.0001, and the specific experimental results are shown in Table 8. In our tests, we maintain uniform parameter settings and discover that the TPSAO significantly outperforms the traditional learning rate of 0.001 in identifying the optimal learning rate for diagnosing pepper diseases. While the SAO and the RIME also have some success in this area, the SAO’s iterative process tends to focus on discovering the next learning rate close to the optimal solution, and although the RIME does not exhibit this behavior, its outcomes are still not as favorable as those achieved by the TPSAO. The unique benefit of the TPSAO method compared to other approaches is its implementation of tent chaotic mapping for initialization, which guarantees a more uniform distribution of initial solutions while incorporating both the position and the velocity update features from the PSO. This dual mechanism enables particles to react to both the immediate optimal and the overall optimal positions, efficiently avoiding the dilemma of accepting suboptimal solutions and facilitating the discovery of the truly optimal learning rate. Despite the TPSAO’s limitations in computational efficiency and adaptability, these points offer valuable insights for future enhancements to further improve the algorithm’s utility and effectiveness.

### 3.5. Ablation Experiment

To validate the effectiveness of the proposed method, we conduct ablation experiments on the TPSAO-AMWNet. We utilize the control variable method to systematically incorporate the ARPC, MTDFA, and WfrLoss modules and explore different combinations of these modules. The findings demonstrate that under identical conditions (Batch_size = 32, learning rate = 0.001, Epoch = 200, Optimizer = Adam), the accuracy incrementally improves with the integration of additional modules. Notably, the configurations combining ARPC + WfrLoss and MTDFA + WfrLoss yield better results than using individual modules separately, underscoring the efficiency and necessity of the integrated approach.

Moreover, we confirm the efficacy of the TPSAO algorithm by employing the control variable method, progressively incorporating the ARPC, MTDFA, and WfrLoss modules in various configuration combinations. The experimental parameters are set as the number of particles *N* = 50, the maximum number of iterations Maxiter = 100, the upper limit of learning rate upper_bound = 0.01, the lower limit of learning rate lower_bound = 0.0001, the Batch_size = 32, Epoch = 200, and the Optimizer = Adam. The experimental findings indicate that the TPSAO effectively identifies the optimal learning rate for each model. When compared to the fixed learning rate approach, the TPSAO demonstrates clear benefits in pinpointing the ideal learning rate, significantly enhancing both the performance and the accuracy of the model. The specific experimental results are shown in Table 9.

### 3.6. Comparison with State-of-the-Art Methods

**Figure 7:**
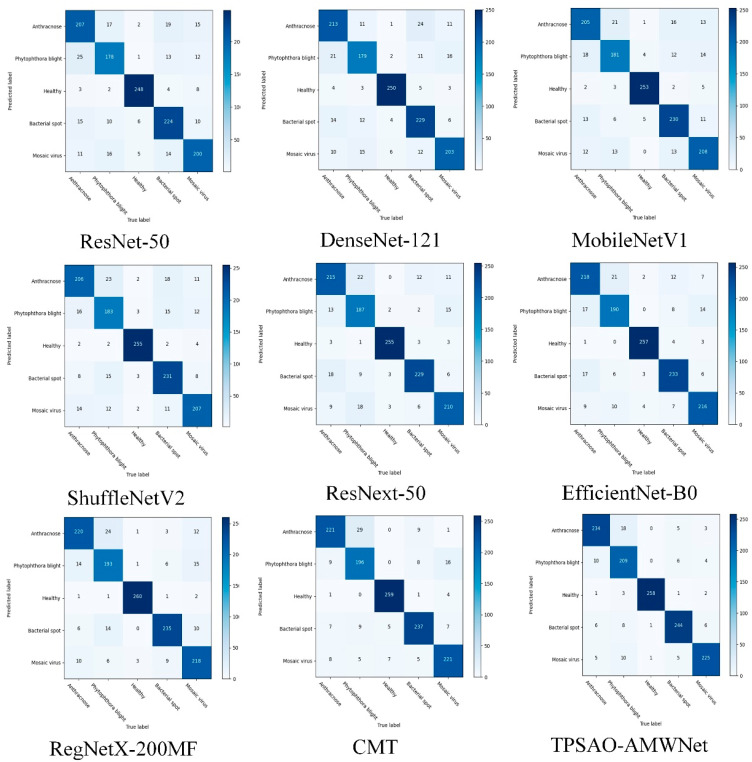
Confusion matrix.

We compare the TPSAO-AMWNet model with classical convolutional neural networks and state-of-the-art networks such as ResNet-50 [51], DenseNet121 [52], MobileNetV1 [53], ShuffleNetV2 [54], ResNeXt-50 [55], EfficientNet-B0 [56], RegNetX-200MF [57], and CMT [58]. In order to ensure the consistency of the experiment, the TPSAO is not used to adjust the optimal learning rate of the TPSAO-AMWNet. Instead, we uniformly set the hyperparameters to be a Batch_size = 32, a Learning rate = 0.001, an Epoch = 200, and an Optimizer = Adam, and we perform the experimental comparison. The confusion matrix, seen in Figure 7, demonstrates the performance of each model in disease recognition, with a high level of accurate recognition for each class. Table 10 shows that the TPSAO-AMWNet has higher recall metrics than Anthracnose and the Phytophthora blight on Healthy, Bacterial spot, and Mosaic virus diseases. This is due to the introduction of the MTDFA module into the basic network of the TPSAO-AMWNet, which can effectively balance the extraction of global and local information and is conducive to the feature extraction of minor diseases, thereby improving its recognition accuracy. Furthermore, the average accuracy of the TPSAO-AMWNet is 92.43%, the highest record among all network models, thereby thoroughly demonstrating the viability of the TPSAO-AMWNet. Figure 7 also shows that the TPSAO-AMWNet has the highest recognition accuracy and the highest number of recognitions.

### 3.7. Generalization Experiments

The generalization experiment serves as a crucial indicator for evaluating model performance. It demonstrates the model’s performance across multiple datasets and reflects its ability to generalize and withstand variations in classification methods. These experiments thoroughly examine the adaptability of the model in handling diverse image qualities, resolutions, and lighting conditions, as well as complex backgrounds of plant leaves and non-plant leaves. This comprehensive evaluation helps us to better understand the applicability and reliability of the model.

Consequently, to more thoroughly assess the effectiveness of the TPSAO-AMWNet, we carry out experiments on widely recognized generalization validation benchmark datasets (such as Flower, PlantVillage grape, Stanford dogs, PlantVillage tomato, and animals). The hyperparameters are configured as follows: Batch_size = 32, Learning rate = 0.001, Epoch = 200, Optimizer = Adam, set = 1, γ = 0 in WfrLoss, which transforms into a binary cross-entropy loss function with L2 regularization added. Table 11 offers comprehensive statistical data information, including the category, data volume, and data source, with all input images being 256 × 256, and the ratio of training to test sets is 8:2. The specific experimental results are shown in Table 12. The TPSAO-AMWNet is weakly identified on the Stanford dog datasets because the datasets have more taxonomic categories and fewer image data in each category. The amount of data cannot meet the requirements of the complex network structure of the TPSAO-AMWNet for image feature learning, resulting in the low recognition accuracy of the TPSAO-AMWNet for these datasets. Since the images in Plantvillage (Grape), Plantvillage (Tomato), and animals have less interference, more obvious target features, and larger data volumes, which can provide good training for the TPSAO-AMWNet, the recognition accuracy of the TPSAO-AMWNet in Plantvillage (Grape), Plantvillage (Tomato), and animals is higher than the remaining two datasets. The identification of the TPSAO-AMWNet is relatively lower than Plantvillage (Grape) and Plantvillage (Tomato) on the Flower datasets because the datasets have many taxonomic categories, and there may be some similarity problems in the categories, resulting in the relatively low identification accuracy of the TPSAO-AMWNet on these datasets. In future studies, we should address the challenge of the similarity between categories, simplify the network structure, and achieve ahigher identification accuracy with smaller amounts of data.

## 4. Discussion

The effectiveness of the TPSAO-AMWNet in the identification of pepper diseases is evaluated and compared with that of ResNet-50 [51], DenseNet-121 [52], MobileNetV1 [53], ShuffleNetV2 [54], ResNeXt-50 [55], EfficientNet-B0 [56], RegNetX-200MF [57], and CMT [58]. We utilize a confusion matrix to more effectively evaluate the recognition capabilities of these various networks, and Figure 7 displays the classification outcomes for the nine networks. From Table 10, we can see that the TPSAO-AMWNet better identifies the five pepper diseases in the datasets than the traditional mainstream networks. ResNet-50 solves the problem of reduced learning efficiency and accuracy due to increasing network depth by adopting a residual block design, and this structure avoids information loss and selective omission in deep networks, which improves the recognition accuracy of the model and ensures smooth data transmission, but its identification precision is only (80.86%, 78.01%, 93.31%, 85.21%, and 83.48%), with an average accuracy of 83.35%. DenseNet-121 adopts a densely connected structure, in which the output of each layer is used as the input to all subsequent layers, which significantly reduces the number of parameters of the network, and effectively solves the problems of gradient disappearance and parameter sparsity. This technique not only boosts the model’s generalization capability but also increases the overall accuracy of the model (82.52%, 78.21%, 94.65%, 86.68%, and 84.03%), with an average accuracy of 84.83%. MobileNetV1, by using deep residual separable convolution to optimize performance and reduce the computing burden, greatly reduces the number of parameters and computations, while maintaining the network performance, and the precision of the model is (79.46%, 78.60%, 95.62%, 86.80%, 85.96%), with the average accuracy of 84.94%. ShuffleNetV2 effectively optimizes the network structure by combining channel shuffle and group convolution, and channel rearrangement allows the features from group convolution to be cross-combined between groups. The method effectively avoids the problem of information isolation and improves the performance of the model. The precision of the model is (78.08%, 80.48%, 94.85%, 86.50%, 84.79%), and the average accuracy is 85.72%. ResNeXt-50 is an improvement of ResNet-50, in which the introduction of group convolution in the residual block further extends the function of the network. ResNeXt-50 learns the different feature subspaces of the input and enhances the robustness of the network. The precision of the model is (82.52%, 81.52%, 96.03%, 87.80%, 86.42%), and the average accuracy is 86.47%. Compared with ResNet-50, the recognition of the five pepper diseases by the network is improved (+1.66%, +3.51%, +2.72%, +2.94%), and the average accuracy is improved by 3.12%; however, these enhancements are accompanied by a substantial rise in the number of parameters and a reduction in recognition efficiency. EfficientNet-B0 uses a compound scaling method, which systematically adjusts the ratio of the depth, width, and input resolution of the network, this well-coordinated scaling approach significantly enhances both the efficiency and the performance of the model, and it solves the common problems of efficiency degradation and performance bottlenecks in the expansion of traditional models. The precision of the model is (83.21%, 82.85%, 96.60%, 87.31%, 87.10%), and the average accuracy is 87.90%. RegNetX-200MF adopts a rule-based network design approach that focuses on achieving uniform and efficient performance by systematically optimizing the width and depth of each layer. This approach markedly diminishes the model’s parameter count and computational demands by meticulously managing the number of channels and the repetition of layers; it effectively balances the problem between efficiency and adaptability. The precision of the model is (84.29%, 83.41%, 96.74%, 88.40%, 88.20%), and the average accuracy is 88.86%. The CMT network integrates the strengths of convolutional neural networks (CNNs) and Transformer technology and enhances the ability of the model to deal with local features and global dependencies by integrating convolution operations into the attention mechanism of the Transformer, with the precision of the model being (85.46%, 85.39%, 97.29%, 89.48%, and 90.13%), with an average accuracy of 89.52%.

In the confusion matrix depicted in Figure 7, the numbers within each square represent the classification results obtained by the classification network for each disease. The color depth of the square represents the accuracy of the classification. The darker the color is, the higher the prediction accuracy of the category, that is, the greater the number of true positives. At the same time, this visualization also helps us to identify in which categories the network performs better or has the problem of misclassification. By comparing the values on the diagonal with the values on the other diagonals, we can intuitively see the classification effect and misclassification of the network in different categories. The comparison of the indicators in Table 10 and the confusion matrix in Figure 7 show that compared with other networks, the TPSAO-AMWNet’s classification is the best, meeting the basic disease identification requirements. In addition, in order to verify the ability of the model to identify various types of other plant leaves, we downloaded the public datasets on PlantVillage, conducted experiments, and achieved good experimental results as shown in Table 12, with a mean accuracy of 96.15%.

This paper summarizes the reasons why the TPSAO-AMWNet outperforms other mainstream networks.

1. In order to highlight the edge of the pepper diseases, this study improves the pyramid convolution and integrates the SE module to emphasize the pepper disease feature image area. As shown in the experimental results presented in Table 5, it is evident that the inclusion of the ARPC structure and pyramid convolution in the model has led to an improvement in the accuracy of pepper disease classification. Specifically, there is a 2.15% increase when compared to the model without the ARPC structure, and a 1.35% increase when compared to the model without pyramid convolution.
2. In order to effectively capture the minor disease characteristics and balance global and local feature extraction, this paper introduces MTDFA. According to the experimental results in Table 6, the accuracy of pepper disease classification is improved by 3.65% after the addition of MTDFA.
3. To cope with the problems of model overfitting and selecting the best learning rate, we add L2 regularization to Weighted Focal Loss and determine the best learning rate using the TPSAO algorithm. Through the experimental data in Table 7 and Table 8, it can be seen that these measures effectively improve the accuracy of pepper disease classification.
4. As shown in the ablation experiments in Table 9, the four modules of our network assume different functions, including solving the edge-blurring problem, extracting tiny features, preventing overfitting, and optimizing the search for the learning rate. The integration of these modules significantly enhances the overall performance of the network and has a clear positive impact on the model’s effectiveness.
5. According to our study, this method has a higher classification accuracy than other deep neural network models, with an average accuracy of 92.43%.

In general, the TPSAO-AMWNet model performs well in classifying pepper diseases and also shows good results in classifying various types of leaves of other plants. Nonetheless, given the large number of pepper diseases and complex leaf characteristics, our experiments failed to adequately address the identification of other diseases and the similarity between different diseases. Current research focuses on disease recognition at the image acquisition stage. To extend this identification process to the full cycle of pepper cultivation and the dynamic large-scale monitoring of diseases, we should explore the potential of the model in real-time applications and its scalability. In addition, it is crucial to ensure that the model operates effectively in real agricultural environments, which requires enhancing the model’s adaptability to various environmental conditions, such as different light intensities, weather variations, and planting backgrounds. Further, combining AI technology with agricultural Internet of Things (IoT) devices allows for automated and real-time disease monitoring; by deploying sensors and cameras in the field, data on potential diseases of chili peppers can be collected continuously; this data can be transmitted in real-time to a cloud platform and analyzed using a trained model to provide a quick response and guide the farmer to take the appropriate measures. In addition, the scalability of such a system is critical; it should be designed to be able to easily add new sensors, etc., to accommodate different crops and disease types, thus broadening its range of applications. It is also necessary to develop user-friendly interfaces that allow agriculturalists to easily manage and interpret the data output from the model, and should include alarm systems, as well as easy-to-operate control functions. Finally, to ensure the widespread adoption of the technology, it is also important to take into account its cost-effectiveness and ensure that the technological solution is both affordable and easy to deploy. This will not only help to modernize the agricultural industry but will also drive its intelligence.

## 5. Conclusions

In this paper, we propose a new TPSAO-AMWNet-based pepper disease recognition method, which optimizes the extraction of tiny disease features and effectively balances global and local features. We also use the TPSAO optimization algorithm to find the optimal learning rate, a step that is less-considered in previous studies, which significantly improves the training effect. To solve the edge-blurring problem and highlight the edges of pepper diseases, APRC is proposed. Meanwhile, we propose MTDFA, which enhances the model’s performance by combining dual-space and dual-channel information to capture the local details of the pepper leaf disease features while maintaining the focus on the global features. In addition, WfrLoss is proposed in this paper, which has a better adaptability, convergence speed, and stability than Weighted Focal Loss and avoids the overfitting problem during the training process. For the difficult problem of determining the optimal learning rate, we use an optimization algorithm to find the most suitable learning rate. Tent chaotic mapping is used to initialize the algorithm, followed by the combination of the SAO and PSO algorithms, and the particles are balanced between the local optimal solution and the overall optimal solution, which effectively optimizes the search process. In the experimental phase, our customized dataset is divided into training and test sets according to an 8:2 ratio. The TPSAO-AMWNet achieves a high recognition accuracy of 93.52% on the test set, which proves the effectiveness of the model. In addition, through modular and ablation experiments, we confirm the effectiveness of each component of APRC, MTDFA, WfrLoss and the TPSAO and their interactions. In generalization experiments, we verify the generalization ability of the model. We also conduct network comparison experiments, which show that our model outperforms other networks and effectively improves disease identification in precision agriculture.

To further optimize the accuracy of pepper disease recognition, future studies will deeply explore specific image preprocessing techniques, such as image enhancement and noise removal, to improve the performance of the model under various lighting and background conditions. In addition, there are plans for future studies to enhance the generalization ability of the deep learning model by expanding the dataset and increasing the experimental scale, and also to integrate transfer learning technology and the pre-trained model into the TPSAO-AMWNet framework to optimize the feature extraction and improve the performance of the model. The implementation of these strategies is expected to significantly improve the efficacy of the model, supporting more efficient disease identification and decision-making in agricultural production.

## Figures and Tables

**Table 1:** Quantity and proportion of pepper leaf diseases.

Disease Category	Number (Before)	Expanded	Number (After)	Proportion/% (After)
Anthracnose	912	384	1296	21%
Phytophthora blight	687	457	1144	18%
Healthy	944	380	1324	21%
Bacterial spot	976	347	1323	21%
Mosaic virus	691	537	1228	19%
Total	4210	2065	6315	100%

**Table 2:** Picture characteristics of pepper leaf diseases.

Disease Category	Pepper Leaf Image		
Anthracnose	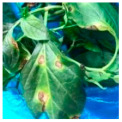	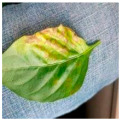	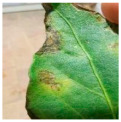
Phytophthora blight	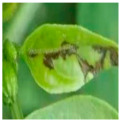	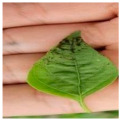	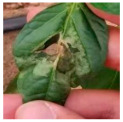
Healthy	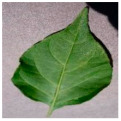	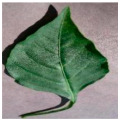	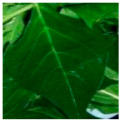
Bacterial spot	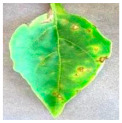	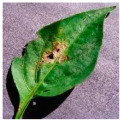	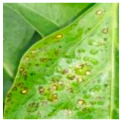
Mosaic virus	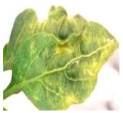	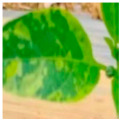	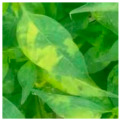

**Table 3:** The software and hardware environments.

Hardware environment	GPUs	RTX 3080×2(20 GB) × 1
	RAM	30 GB
	CPU	7 vCPU Intel(R) Xeon(R) CPU E5-2680 v4 @ 2.40 GHz
	Hard disk	System disk: 30 GB Data disk: 50 GB
Software environment	OS	Windows 10
	PyTorch	1.11.0
	Python	3.8 (ubuntu20.04)
	Cuda	11.3

**Table 4:** The experimental settings.

Hyperparameters	Value
Size of input images	256 × 256
Batch_size	32
Epoch	200
Initial learning rate	0.001
Optimizer	Adam

**Table 5:** The experimental results on the effectiveness of ARPC.

Methods	Precision	Recall	F1	Accuracy
None	86.86	86.29	86.57	86.47
Pyramid convolution	87.23	87.41	87.32	87.27
APRC	88.55	88.36	88.46	88.62

**Table 6:** The experimental results on the effectiveness of MTDFA.

Methods	Precision	Recall	F1	Accuracy
None	86.86	86.29	86.57	86.47
HaloAttention	88.24	88.72	88.48	88.38
CBMA	88.03	88.28	88.16	88.14
Polarized Self-Attention	88.74	88.53	88.64	88.54
MTDFA	90.24	90.18	90.21	90.12

**Table 7:** The experimental results on the effectiveness of WfrLoss.

Methods	Precision	Recall	F1	Accuracy
Ploy Loss	86.65	86.37	86.51	86.26
Cross Entropy	86.86	86.29	86.57	86.47
WfrLoss	87.09	87.15	87.12	87.11

**Table 8:** The results of the TPSAO effectiveness experiments.

Methods	Precision	Recall	F1	Accuracy	Learning Rate
None	86.86	86.29	86.57	86.47	0.001
SAO	88.05	87.93	87.99	87.91	0.003902
RIME	87.41	87.67	87.54	87.59	0.004424
TPSAO	88.56	88.20	88.38	88.22	0.003206

**Table 9:** The results of the ablation experiments.

Methods	Precision	Recall	F1	Accuracy	Learning Rate
ARPC	88.55	88.36	88.46	88.62	0.001
MTDFA	90.24	90.18	90.21	90.12	0.001
WfrLoss	87.09	87.15	87.12	87.11	0.001
TPSAO	88.56	88.20	88.38	88.22	0.003206
ARPC + MTDFA	91.27	91.31	91.29	91.23	0.001
ARPC + WfrLoss	90.02	89.96	89.99	89.72	0.001
MTDFA + WfrLoss	90.47	90.43	90.45	90.51	0.001
ARPC + MTDFA + WfrLoss	91.64	91.50	91.57	91.62	0.001
APRC + TPSAO	90.12	90.05	90.09	90.04	0.003116
MTDFA + TPSAO	91.10	91.06	91.08	91.15	0.002798
APRC + WfrLoss + TPSAO	90.85	90.63	90.74	90.83	0.004036
MTDFA + WfrLoss + TPSAO	91.69	91.61	91.65	91.70	0.003665
ARPC + MTDFA + TPSAO	92.07	92.30	92.19	92.17	0.002249
ARPC + MTDFA + WfrLoss + TPSAO	93.34	93.48	93.41	93.52	0.001914

**Table 10:** A comparison of the experimental results.

Methods	Index	Pepper Disease				
		Anthracnose	Phytophthora Blight	Healthy	Bacterial Spot	Mosaic Virus
ResNet-50	Precision (%)	80.86	78.01	93.31	85.21	83.48
	F1 (%)	80.24	77.87	93.94	84.99	82.58
	Recall (%)	79.62	77.73	94.57	84.78	81.70
	Accuracy (%)	83.35%				
DenseNet-121	Precision (%)	82.52	78.21	94.65	86.68	84.03
	F1 (%)	81.71	78.58	94.57	85.53	83.27
	Recall (%)	80.92	78.96	94.48	84.40	82.52
	Accuracy (%)	84.83%				
MobileNetV1	Precision (%)	79.46	78.60	95.62	86.80	85.96
	F1 (%)	79.15	79.38	95.80	86.28	85.65
	Recall (%)	78.85	80.18	95.98	85.76	85.34
	Accuracy (%)	84.94%				
ShuffleNetV2	Precision (%)	78.08	80.48	94.85	86.50	84.79
	F1 (%)	78.38	80.03	95.40	86.83	84.47
	Recall (%)	78.68	79.59	95.95	87.17	84.15
	Accuracy (%)	85.72%				
ResNeXt-50	Precision (%)	82.52	81.52	96.03	87.80	86.42
	F1 (%)	81.60	82.07	96.20	87.11	85.89
	Recall (%)	80.69	82.63	96.36	86.42	85.37
	Accuracy (%)	86.47%				
EfficientNet-B0	Precision (%)	83.21	82.85	96.60	87.31	87.10
	F1 (%)	83.53	83.35	96.82	87.61	87.45
	Recall (%)	83.85	83.86	97.05	87.92	87.80
	Accuracy (%)	87.90%				
RegNetX-200MF	Precision (%)	84.29	83.41	96.74	88.40	88.20
	F1 (%)	84.46	83.98	96.99	88.74	88.02
	Recall (%)	84.62	84.55	97.24	89.08	87.84
	Accuracy (%)	88.86%				
CMT	Precision (%)	85.46	85.39	97.29	89.48	90.13
	F1 (%)	85.24	86.12	97.67	89.88	89.44
	Recall (%)	85.03	86.86	98.05	90.29	88.76
	Accuracy (%)	89.52%				
TPSAO-AMWNet	Precision (%)	89.71	91.08	97.85	93.27	92.09
	F1 (%)	90.12	91.42	97.95	93.45	92.82
	Recall (%)	90.54	91.76	97.62	93.62	93.57
	Accuracy (%)	92.43%				

**Table 11:** The details of the public datasets.

Dataset	Total	Category	Available (accessed on 10 April 2024)
Flower	13,740	14	https://www.kaggle.com/datasets/marquis03/flower-classification
PlantVillage Grape	4062	4	https://www.kaggle.com/datasets/hiyash99/plantvillage
Stanford dogs	20,580	120	https://www.kaggle.com/datasets/jutrera/stanford-car-dataset-by-classes-folder
PlantVillage Tomato	18,160	10	https://www.kaggle.com/datasets/hiyash99/plantvillage
animals	26,179	10	https://www.kaggle.com/datasets/alessiocorrado99/animals10

**Table 12:** The results of the generalizability experiments.

Dataset	F1 (%)	Accuracy (%)
Flower	96.07	96.56
PlantVillage grape	98.28	98.40
Stanford dogs	90.78	91.14
PlantVillage tomato	96.94	97.49
animals	96.60	97.15

## Data Availability

The datasets used in this paper are available on demand.
